# 4-PHENYLBUTYRATE: MOLECULAR MECHANISMS AND AGING INTERVENTION POTENTIAL

**DOI:** 10.1093/geroni/igz038.379

**Published:** 2019-11-08

**Authors:** Michael R Bene, Kevin Thyne, Jonathan Dorigatti, Adam B Salmon

**Affiliations:** 1 University of Texas Health Science Center at San Antonio, San Antonio, Texas, United States; 2 University of Texas Health at San Antonio, San Antonio, Texas, United States

## Abstract

4-Phenylbutyrate (PBA) is a FDA approved drug for treating patients with urea cycle disorders. Additionally, PBA acts upon several pathways thought of as important modifiers of aging including: histone deacetylation, proteostasis as a chemical chaperone, and stress resistance by regulating expression of oxidative stress response proteins. PBA has also been shown to extend lifespan and improve markers of age-related health in Drosophila. Due to its wide range of effects PBA has been investigated for use in numerous age-related disorders including neurodegenerative and cardiovascular diseases. To better understand the effects of PBA on the molecular level, we used both in cellulo and in vivo studies. Treatment of primary mouse fibroblasts, C2C12 mouse muscle cells, and NCTC 1469 mouse liver cells with PBA demonstrated differential responses among cell lines to upregulation of oxidative stress response and histone acetylation. Specifically, upregulation of the oxidative stress response protein DJ-1 by PBA was found to have a corresponding dose response curve to histone H3 acetylation in primary fibroblasts. To study effects of PBA in vivo, four cohorts of HET3 mice were treated with PBA at different doses in drinking water for 4 weeks. PBA was well tolerated and led to different effects on body composition dependent on the sex of mice. We are currently investigating the molecular effects of PBA treatment in multiple tissues samples from these mice. The potential of PBA to alter many fundamental pathways, and specifically those related to stress responses, make it an attractive prospect for treatment of many age-related disorders.

